# Asia Partnership Conference of Pharmaceutical Associations (APAC) Report on Regulatory Agility Implemented During the COVID-19 Pandemic: Inspiring Partnerships and Recommendations for the Way Forward

**DOI:** 10.1007/s43441-022-00435-8

**Published:** 2022-09-29

**Authors:** Sannie Siaw Foong Chong, Masaaki Kanno, Alice Seat Mee Chee, Siew Mei Long, Stephanie Hui Min Ong, Usanee Harnpramukkul, Richard Simon R. Binos

**Affiliations:** 1 Pharma Technical Regulatory Policy and International Operations, Roche Singapore Technical Operations, F. Hoffmann La-Roche Ltd., Singapore, Singapore; 2 Present Address: Global Regulatory Policy, Global Regulatory Affairs and Clinical Safety, Merck Research Laboratories, MSD, Singapore, Singapore; 3 Overseas Regulatory Office, Regulatory Affairs Department, Otsuka Pharmaceutical Co., Ltd., Tokyo, Japan; 4 Pharmaceutical Association of Malaysia, Petaling Jaya, Malaysia; 5 Merck Sharp & Dohme, Petaling Jaya, Malaysia; 6 Sanofi, Petaling Jaya, Malaysia; 7 Global Regulatory Affairs – International, Pfizer, Bangkok, Thailand; 8 Pharmaceutical and Healthcare Association of the Philippines, Makati, Philippines

**Keywords:** Partnership, Regulatory agility, COVID-19, APAC, NRAs

## Abstract

**Purpose:**

Asia Partnership Conference of Pharmaceutical Associations (APAC) promote regulatory agility of four important best practices i.e. reliance, digital platform, accepting electronic document and process integration. Dialogues and strong partnership witnessed reforms and efficiencies amidst the pandemic. In tracking the progress of regulatory agility, APAC identifies areas for improvement and recommends prioritizing these areas for change.

**Methods:**

As one voice, 13 main industry associations under the umbrella of APAC sent joint letters to our National Regulatory Authorities (NRAs) with a call to maintain regulatory agility and consider new ways of working. Consequently, APAC surveyed its member associations to measure regulatory agilities implemented by the NRAs during 2020 and 2021 in view of the pandemic.

**Results:**

This paper reports progress in implementing regulatory agility, e.g. the number of economies that can accept electronic Certificate of Pharmaceutical Products (eCPP) has reached 100% for the economies that require CPP and more than 90% can waive onsite inspection in the presence of Good Manufacturing Practice (GMP) certificate and/or inspection report. The paper also features the progress made in Malaysia, the Philippines, and the ASEAN (Association of South East Asian Nations) regional reliance initiative to reduce inefficiencies and duplications.

**Conclusions:**

We have demonstrated the power of working together to enable regulatory agilities and efficiencies. APAC will continue to track the progress of all economies including India within the areas for improvement prioritized and discussed in this paper. APAC is also committed to working with key stakeholders including our NRAs in Asia to sustain and enable a new era of innovation ushered in by COVID-19 to benefit patients.

## Background

APAC^1^ is an industry-driven initiative led by research and development (R&D)-based pharmaceutical associations, aiming to fulfill its mission of expediting the launch of innovative medicines for the people in Asia.

In the second half of 2020, APAC recognized the challenges posed by the ongoing COVID-19 pandemic to our NRAs. To ensure the continuity of supply of medicines and treatment to patients, APAC’s 13 main industry associations sent a joint letter to the NRAs in Asia in July 2020 to work in partnership and consider new ways of working.

This is the first time, all the member associations, except the Organization of Pharmaceutical Producers of India (OPPI) under the umbrella of APAC have collectively communicated our objectives as one voice in a form of a joint letter to commend the efforts of NRAs in practicing regulatory agility. Accordingly, APAC recommends four important best practices:

1. Collaborative, coordinated scientific assessment leveraging on reliance practices;
2. Enhanced use of digital platform for communication;
3. Acceptance of electronic document; and
4. Integrate and streamline regulatory processes.^2^

**Diagram 1:**
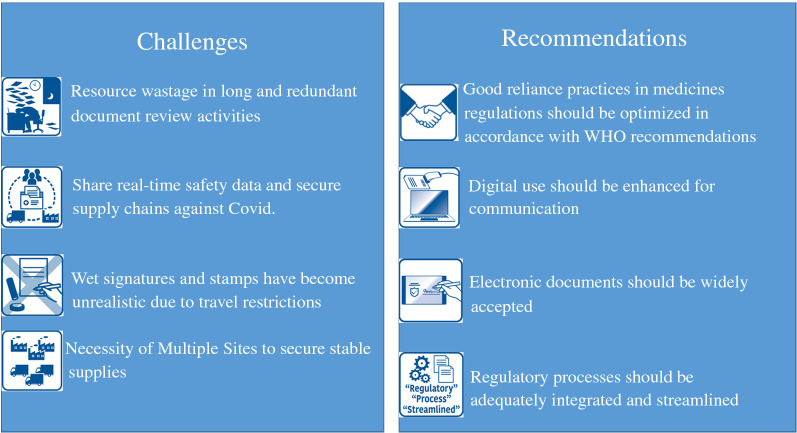
Examples of Challenges and APAC’s recommendations in implementing regulatory agilities.

To ensure efficiency and avoid duplicative review, APAC recommends good reliance practices by leveraging stringent work already conducted by leading NRAs as shown in Diagram 1. The use of digital platforms (e-labeling, traceability of serialization) improves the accessibility of approved medical information and helps in securing pharmaceutical supply chains against counterfeiting. On the other hand, shifting the requirement from wet signatures and legalization to electronic-based certification ensures the continuity of the regulatory processes by eliminating physical steps in the process. Lastly, integrating and streamlining the regulatory processes including multiple production sites in one license facilitates supply flexibility while eliminating duplicative activities.

Following the letter, APAC member associations worked with the respective NRAs towards implementing the recommendations. On 13 April 2021, we the industry associations met with the NRAs in the annual APAC meeting.^3^ We decided to present a joint Thank You letter to all our regulators in Asia after the APAC meeting. In June to July 2021, APAC sent a follow-through letter^4^ to thank the regulators for taking action and practicing regulatory agility during global crisis; as well as to highlight regulatory processes that remain challenging for patients’ access. To determine the progress and response of the NRAs to the recommendations mentioned in the letters, APAC decided to measure the status of regulatory landscape of our NRAs.

## Methodology

In July 2020 and December 2021, the APAC conducted a survey to measure the implementation of regulatory agilities recommended by APAC in all 11 economies in Asia.

### Survey Participants

Apart from OPPI, the survey was sent to all the 13 APAC member associations. Since there are two member associations in China and South Korea, the responses from the same economies were considered as one valid response to avoid duplication.

## Materials

A progress status form was prepared. It contained question-–response type of questionnaires that consisted of four sections with questions designed to determine the level of regulatory agility practiced by the NRA. The progress status form can be found at the link.^5^

### Procedure

#### Preparing the Letters

Prior to the distribution of the APAC letter to the respective NRAs, signature of each member association was required. All members reviewed and signed the letter except OPPI. Consequently, APAC did not send the letters to the NRA of India. The APAC secretariat sent the letters to the NRAs through the names and emails provided by the member associations. The member associations were included in the same email in case of clarifications or follow-up actions.

The recipients of the APAC letters were:

Box 1: Recipient NRAs of APAC Letters

**Table:** 

Economies	Abbreviations	National Regulatory Authority (NRA)
Vietnam	VN	Drug Administration of Vietnam (DAV)
Hong Kong	HK	Drug Office, Department of Health (DOH)
Singapore	SG	Health Sciences Authority (HSA)
Indonesia	ID	National Agency of Drug and Food Control (BPOM)
China	CN	National Medical Products Administration (NMPA)
Malaysia	MY	National Pharmaceutical Regulatory Agency (NPRA)
South Korea	SK	Ministry of Food and Drug Safety (MFDS)
Japan	JP	Pharmaceuticals and Medical Devices Agency (PMDA)
Philippines	PH	Philippines Food and Drug Administration (PFDA)
Taiwan	TW	Taiwan Food and Drug Administration (TFDA)
Thailand	TH	Thailand Food and Drug Administration (Thai FDA)

#### Collecting the Data

Data collection commenced in the second half of 2020, with reference to the direct response of the government officials to the letters or from the industry associations.^6^ Official replies from Taiwan FDA^7^ and HK MOH were received in later part of 2020. To obtain feedback, APAC member associations also conducted dialogue sessions to follow-up with our NRAs.

To track progress, APAC conducted a survey to all APAC members on 24 August 2021 and received responses from member associations in China, Hong Kong, Indonesia, Japan, Malaysia, Philippines, Singapore, South Korea, Taiwan, Thailand, and Vietnam.

When needed, APAC also referred to peer-reviewed publications [1], official websites, APAC analysis reports^8^ or contacted member associations directly for collecting retrospective baseline data (prior to COVID-19 pandemic).

## Results

### Market Authorization Agilities/Reliance Including Inspection, Import Re-Testing and Post-Approval Agilities

**Fig. 1:**
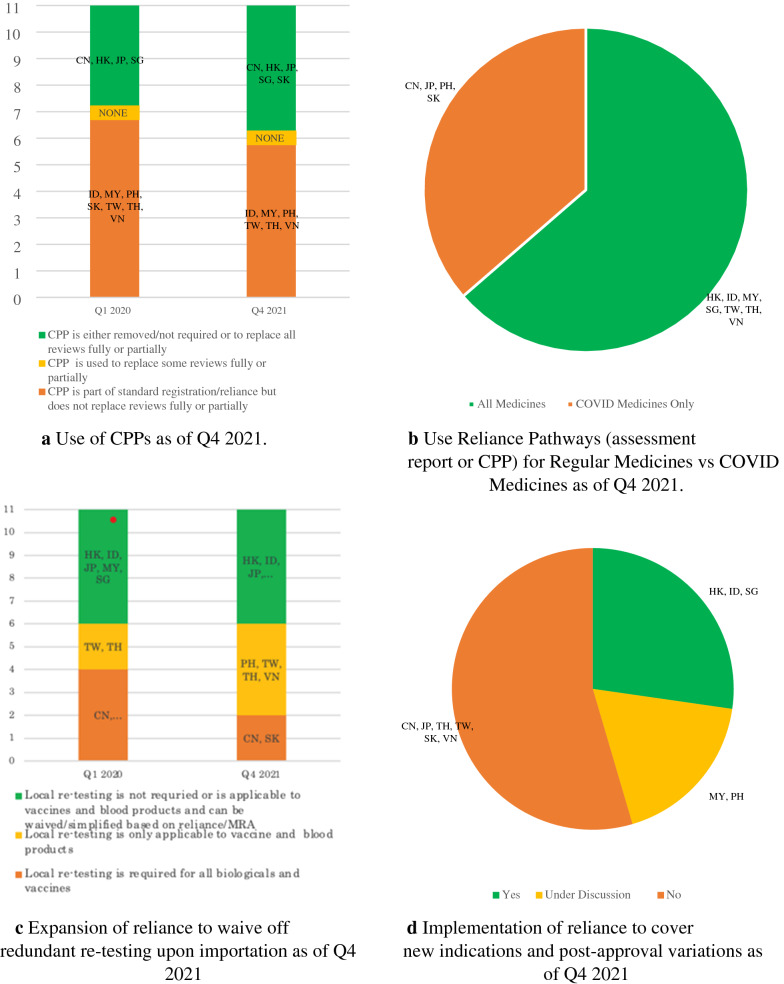
Implementation of reliance as of Q4 2021

CPP is part of the registration pathways including reliance pathways in Indonesia, Malaysia, Philippines, South Korea, Taiwan, Thailand, and Vietnam as shown in Fig. 1a. Different from its original intent, CPP is required while these NRAs continue to conduct full evaluation. On the other hand, Hong Kong used CPP to replace full or partial review as defined by the World Health Organization (WHO) [2]. It is worth noting that China, Japan, and Singapore do not require CPP though companies may choose to use CPP in Singapore as proof of approval. Recently, South Korea too removed CPP from its registration requirement [3]. As of Q4 2021, Hong Kong has one standard pathway that relies on CPP, while reliance pathways exist in Indonesia, Malaysia, Singapore, Taiwan, Thailand, and Vietnam based on assessment report or CPP of the reference agencies for evaluation of all products. Although reliance pathway is not available for general product registration in China, Japan, Philippines, and South Korea, these economies may leverage reliance practice to approve COVID products as shown in Fig. 1b.

Import re-testing is applicable in all the economies for vaccines and some biologics (e.g. blood products) as shown in Fig. 1c; of which Hong Kong, Indonesia, Malaysia, and Singapore may conduct desktop review and/or leverage reliance of reference agencies’ approval to fulfill re-testing assessment. The Philippines revised import re-testing requirement in 2021 from applicable to all biologics to applicable to vaccines and some biologics only. This is similar to that practiced by most of the ASEAN countries [4]. Similarly, the requirement in Vietnam also applies to vaccines and some biologics by Q4 2021 [5]. Japan can waive off import re-testing based on Mutual Recognition Arrangement (MRA) while this arrangement is currently absent in China and South Korea.

As of Q4 2021, Hong Kong, Indonesia, and Singapore apply reliance to post-approval variations and indications while this is under discussion in Malaysia and Philippines as shown in Fig. 1d.

## Digital Platform (e-Labeling, Traceability or Serialization)

**Fig. 2:**
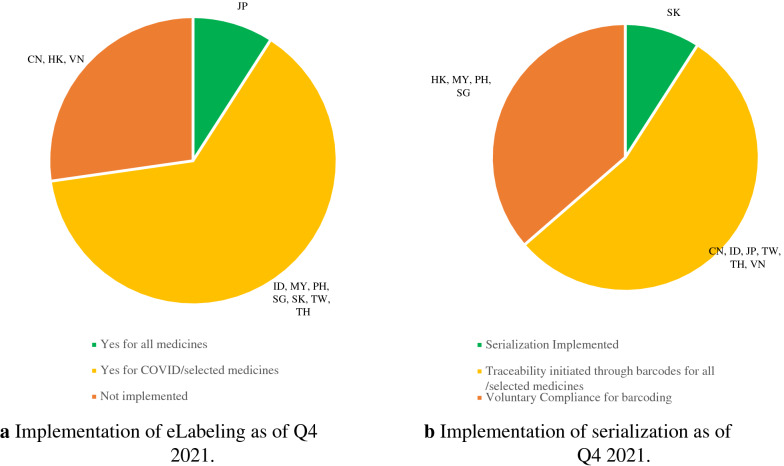
Implementation of digital platform as of Q4 2021

As of Q4 2021, Japan implemented e-labeling [6], while Indonesia, Malaysia, Philippines, Singapore, South Korea, Taiwan, and Thailand fully adopt it for COVID-19 products. China, Hong Kong, and Vietnam have yet to implement e-labeling as shown in Fig. 2a. Figure 2b shows full implementation of serialization in South Korea as of Q4 2021. China, Indonesia, Japan, Taiwan, Thailand, and Vietnam initiated traceability through barcodes or QR codes, whereas the implementation in Hong Kong, Malaysia, Philippines, and Singapore is on a voluntary compliance.

## From Paper to Electronic-Based Document

**Fig. 3:**
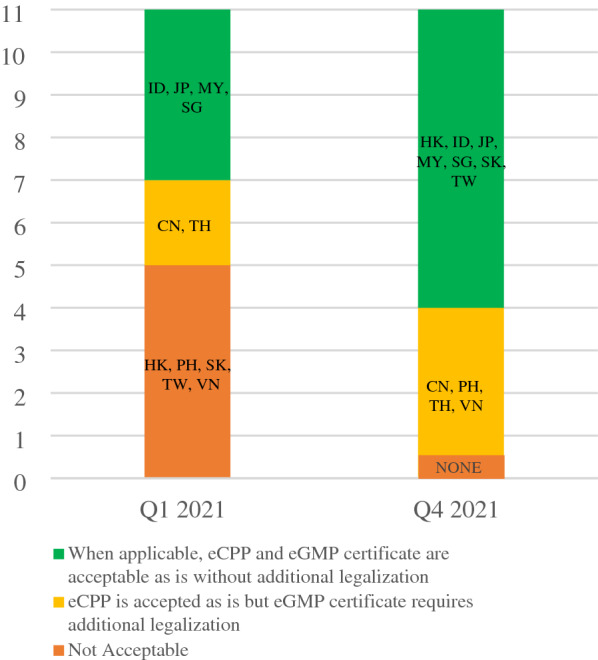
Acceptability of eCPP and eGMP Certificate as of Q4 2021. For post-approval variations, Hong Kong may still require additional legalization for eGMP certificate, https://www.drugoffice.gov.hk/eps/do/en/doc/guidelines_forms/copGuide.pdf?v=lvxgyq. [35]

In Q1 2021, CPP is not required in Japan and Singapore; and both economies accepted electronic GMP (eGMP) certificate when applicable. Indonesia and Malaysia accepted eCPP and eGMP certificate. China and Thailand accepted eCPP as is but require additional legalization for eGMP certificate. By Q4 2021, all economies accepted eCPP as is. However, China, Philippines, Thailand, and Vietnam require additional legalization on eGMP certificate as shown in Fig. 3.

## Integrating and Streamlining Processes (e.g. Multiple Sites in One License/Non-site-specific Stability Data)

**Fig. 4:**
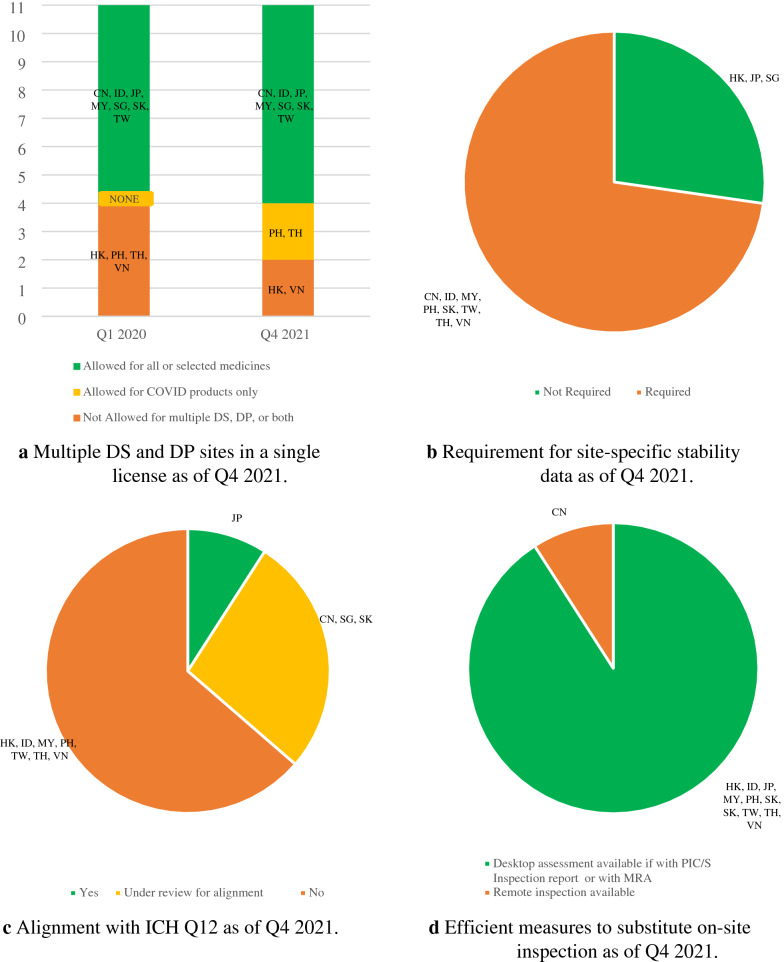
Streamlining and integrating processes as of Q4 2021

In Q1 2020, China, Indonesia, Japan, Malaysia, Singapore, South Korea, and Taiwan practiced multiple sites in a single license. Among them, some economies apply multiple sites in a single license for specific procedures and product categories only and not for all the evaluation pathways. By Q4 2021, both Philippines and Thailand also accepted multiple sites in a single license for COVID products as shown in Fig. 4a. Hong Kong only allows a single drug substance (DS) and a single drug product (DP) manufacturer in one license [7].

Site-specific stability data remains largely a challenge in Asia as shown in Fig. 4b. On the contrary, this is not a requirement in Hong Kong, Japan, and Singapore. As of Q4 2021, Japan aligns with Q12 [8] of the ICH as shown in Fig. 4c. The alignment to ICH Q12 is currently under review in China, Singapore, and South Korea.

All the NRAs implemented efficient measures as of Q4 2021 to substitute on-site inspection as shown in Fig. 4d. China has adopted remote inspection [9, 10], but efficiency as measured by utilization of PIC/S GMP inspection reports or with a MRA is still under exploration.

## Discussion

### Progress in Implementing Regulatory Agility, and Areas to Prioritize Improvements Include:

#### Market Authorization Agilities/Reliance

There continues to be a long review and approval process in economies that require CPP. APAC recommends removing the CPP when it does not replace full or partial assessment based on its original intent defined by the WHO [2, 11, 12]. This will eliminate resource wastage in redundant document review activities. The NRAs have made great strides toward approving COVID-19 products by leveraging assessments performed by the reference agencies in reaching their own decisions within a shorter timeline than the standard procedures. APAC recommends integrating approval process based on reliance into the system to approve all product types and all submission applications, including line extensions and post-approval variations. Removing or reducing import re-testing based on reliance is a welcome development. APAC recommends retaining this agility and operationalizing beyond COVID-19 products. Aligned with the COVID-19 registration experience, the Emergency Use Authorization (EUA) pathway demonstrates how the expedited pathway can work in reality, whereby reliance on stringent regulatory authorities helped facilitate the process [13]. NRAs can leverage the processes and procedures gained from EUA to develop future expedited pathways moving forward.

#### Digital Platform (e-Labeling, Traceability or Serialization)

Japan has implemented e-labeling with the availability of electronic product information (ePI). Serialization is fully implemented in South Korea, although ePI is yet to be made available for all products. Nevertheless, for COVID-19 products all NRAs except China, Hong Kong and Vietnam have implemented ePI. Traceability is initiated in all economies with some on a voluntary compliance. Traceability and ePI ensure faster deployment of product information and improves health system resilience. The International Federation of Pharmaceutical Manufacturers and Associations (IFPMA) supports the use of globally harmonized and standardized two-dimensional (2D) barcodes to provide a direct link from packaging to electronic product information (e.g., 2D barcodes based on International Organization for Standardization / International Electrotechnical Commission (ISO/IEC) 16022 Data Matrix barcode standard) [14].

#### From Paper to Electronic-Based Document

Economies that require CPP can accept eCPP as is. In the case of eGMP certificate, additional steps in legalization including embassy stamps and wet signatures are required. APAC recommends removing these legalization processes since checking and verifying electronically is feasible.

#### Integrating and Streamlining Processes (e.g. Multiple Sites in One License/Non site-Specific Stability Data)

ICH and WHO guidelines classify adding a manufacturing site as a post-approval variation, resulting in multiple sites in one product license [15, 16]. In contrast, site addition approved as a new drug application will result in one site in one license and consequently the need for site-specific stability data. This approach complicates and hinders the addition of multiple sites, without enhancing the value of regulatory oversight [1]. The recommendation is to adopt the practice of multiple sites in a single license, in line with ICH and WHO guidance. Waiver of site-specific stability studies on multiple drug substance and product sites need to be considered or batches reduced to ensure timely and stable supplies, and these best practices are essential to ameliorate the impact of public crisis on the availability of medicines including vaccines. Singapore removed site-specific stability data in 2020 [17], a reference that NRAs could potentially leverage to make the same change in reducing site-specific stability data.

**Fig. 5:**
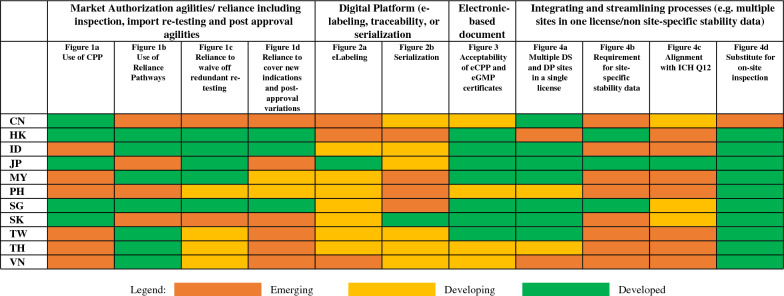
Areas to prioritize improvement based on status in Q4 2021

ICH and PIC/S are platforms for disseminating regulatory information and for building knowledge and trust among NRAs. Japan is exemplary in embracing international guidelines including ICH Q12 [1]. Likewise, all NRAs should rigorously implement harmonized ICH principles of Q12 as regulatory standards to facilitate the review and provide a single technical basis for approval. As shown in Fig. 5, there exist efficient measures to substitute on-site GMP inspection, including the adoption of remote inspection. It is envisaged that these measures under COVID can become a long-term approach and can be formulated into the process of business-as-usual moving forward [18].

### The Importance of Inspiring Partnership

#### Case Study: The Philippines FDA’s Reforms Amidst the Pandemic

The Philippines FDA (PFDA) was under new management just months prior to the pandemic. This reopened communication lines between regulators and industry stakeholders. To improve regulatory services, the PFDA presents reform plans and gathers industry concerns and suggestions through the reinstitution of the quarterly dialogue. Consultative and/or pre-submission meetings with individual companies were allowed on a case-to-case basis, and opportunities for discourse on regulatory findings (e.g. deficiencies, disapprovals) were considered. PFDA, in consultation with the industry, started revisiting existing standards and policies, welcomed inputs from regional and global associations such as APAC and participated in conferences and fora. The collaborative mindset helped PFDA and the Philippines as a whole to better manage the then-approaching crisis.

While the Philippines was described as having the “longest” and “strictest” lockdown in the world, the partnership continued and grew stronger. In the last two years, the PFDA has worked on regulatory policies that address concerns raised by the industry. The PFDA drafted policies and consulted with the industry, while the industry prepared suggestions and recommendations for its improvement by highlighting international best practices and standards. Among other, these reforms include: (1) the shift of focus to risk management plans and global post-marketing surveillance activities [19]; (2) limiting lot release certification requirements to vaccines, toxoids, and immunoglobulins [4]; (3) acceptance of electronic signatures, and eCPP; (4) digitalization of certain regulatory processes [20–22]; and (5) introduction of facilitated registration procedures through reliance [23, 24].

Regulatory reliance was among the reforms that were fast-tracked by the pandemic experience. In order to make available the latest COVID-19-related technologies (e.g. diagnostics, vaccines, and therapies) at the earliest time possible, the PFDA needed an alternative registration scheme that relied on the reviews already conducted by stringent regulatory authorities. The EUA pathway was instituted which relied on assessment reports issued by identified regulatory authorities. The EUA scheme served as a learning experience for both regulators and participating industry stakeholders, and it made both parties realize that the Philippine industry is capable to be at par with regulations of neighboring Asian countries [25]. As a result, Philippines explored other agilities, including: (1) multiple sites under a single license; (2) non-site-specific stability reports; and (3) “smart” or electronic labeling.

Although some of the agilities initiated are yet to be formalized into policy, the experience of regular exchange between parties helped in ensuring that regulatory interventions were quick and targeted, especially as companies strive to bring COVID-19 related technologies to Filipino patients. APAC anticipates that the experience of working in this new framework will accelerate further reforms—a feat that was not possible before.

Box 2: The Philippines case study **The Philippines Case Study: Gaps and recommendations**

• Implementation of Regulatory Reliance. Reliance registration schemes have recently been finalized[36] for non-COVID drugs. While inputs have been provided and incorporated, we are yet to see the actual implementation and its impact.

• Availability of e-labeling. There is no formal policy yet allowing the use of e-labels. A pilot study needs to be conducted and revisions in the existing policy – that requires paper labeling information – be instituted.

• Multiple sites in one license. The FDA requires “one-site-one-license” for all drug products, making it difficult to arrange alternative sources for the same product, especially during shortages. Policy revision is necessary, taking note of the best practices from other NRAs.

### The Outcome of Partnership in Advancing Regulatory Transformation in Asia

#### Case Study: Malaysia NPRA at the Forefront of Digitalization and Modernization

There is an increasing need for regulators to innovate and collaborate to inspire impact, facilitate, and sustain earlier access of medicinal products to patients. The National Pharmaceutical Regulatory Agency of Malaysia (NPRA) has been exemplary in leading system modernization particularly in the areas of reliance, digitalization, and access to innovation.

Following the success of the seven participating NRAs jointly reviewing the anti-malaria drug in 2017 facilitated by WHO, Malaysia published the ASEAN Joint Assessment (AJA) Procedure Guideline and ASEAN Priority Disease List in 2020 [26]. NPRA received a proposition from the Pharmaceutical Association of Malaysia (PhAMA) to improve and optimize the AJA and the proposal was brought up to the ASEAN Joint Assessment Coordinating Group (JACG) for consideration [27]. These recommendations derived from leveraging best practices observed from successful international collaborations such as ACCESS Consortium [28] and Project ORBIS [29], which includes but not limited to (a) expanding the eligibility list to all diseases and product types; and (b) streamlining the administrative procedures to improve the efficiency and pace of the work sharing initiatives among the ASEAN countries. The optimization of AJA will encourage greater industry participation and unleash the true potential of AJA benefiting both regulators and industry alike—leading to earlier regulatory access of innovative medicines to patients. At the 6th JACG meeting on 28–29 October 2021, it has since been agreed to enhance the AJA procedure with the aim to overcome the challenges observed, to clarify steps in the AJA process and timeline, to update the Frequently Asked Questions (FAQ) document and to expand the list of priority products. The AJA Guideline and ASEAN Priority Disease List have been updated to incorporate many of the recommendations.

Akin to global regulators placing prominent emphasis on regulatory reliance, the NPRA is looking at piloting a project to utilize the assessment reports from other reference agencies in addition to the European Medicines Agency (EMA) and the US FDA in consideration of expanding the scope of the facilitated review pathway to maximize the potential of reliance. Additionally, Malaysia is also exploring and considering concepts of reliance for post approval product quality lifecycle by collecting data to see feasibility of including this process in future Malaysian Variation Guideline [30] revisions. Malaysia with the assistance from PhAMA is also open to further understand the Post Approval Change Management Protocol (PACMP) and ICH Q12 [15] on the implications of the variation reviews and approvals.

In the aspect of digitalization, the Ministry of Health (MOH) Malaysia is fully supportive of the initiatives and is looking to expedite the adoption of e-labeling in Malaysia and the possible alignment to the planned implementation of serialization in 2023. A joint Industry-MOH working group led by MOH has been formed to work further on the proposal. The collaborative efforts by the team will include a roadmap review, stakeholder mapping, survey on acceptability of e-labeling in Malaysia, and other necessary actions.

NPRA is moving towards modernization of regulatory submission systems to ensure the highest level of work efficiencies to drive better outcomes for patients. Accordingly, Malaysia has made a commitment to prioritize the optimization of the online system QUEST as an important measure to address industry’s concerns. In particular, with the planned system upgrade, NPRA has committed to explore the possibility to allow the submission of multiple sites on a single license for products and parallel/multiple submissions of additional indication applications. In scope to pave ways for innovative pathways or strategies in Malaysia, Real World Data/Evidence (RWD/E) capacity building with the introduction of the concept to facilitate decision-making, as well as increasing awareness and understanding of the implementation of RWD/E in regulatory decision-making are in progress. RWD/E has the potential to accelerate the delivery of safe and cost-effective therapeutic interventions and to support the expansion of approved therapeutics through a new indication, formulation, and label expansion [31].

Box 3: Malaysia case study **Malaysia case study:** Gaps and recommendations

ASEAN Joint Assessment:

• Gap: Need for process optimization and increased transparency (including on timelines).

• Recommendation: Political commitment, appropriate governance structures and funding, and well-functioning project management with adoption of global best practices are key to support its implementation.

Fostering Reliance:

• Gap: Need to further maximize the potential of reliance.

• Recommendation: To expand the scope of facilitated review pathway including for post approval product quality lifecycle management.

Digitalization (E-labeling):

• Gap: Cost to acquire and build supplementary technological system to support e-labeling and communication to facilitate the implementation of e-labeling and future enhancement.

• Recommendation: Planning and harmonizing labeling information to comply with local regulations along with effective collaboration between the industry and regulators to design a robust proposal for Malaysia.

Modernization of Regulatory Submission:

• Gap: Lack of expertise and inadequate experience in the selected field needs to be improved for better work efficiencies and modernized approaches to drive better health outcomes for patients.

• Recommendations: Optimization of the online QUEST system to support all types of regulatory submissions; and capacity building on the use of Real-World Data/Evidence (RWD/E) to facilitate regulatory decision-making, and the development of an RWE regulatory framework.

## Conclusion

### Improving in Key Areas

APAC will continue to track the progress of all economies including India within the areas for improvement prioritized and discussed in this paper.

### Supporting a Regional Reliance Program

ASEAN will be implementing ASEAN Pharmaceutical Regulatory Policy (APRP) [32] to align with principles of international standards and best practices, eliminate country-specific requirements and promote the concepts of reliance and MRAs. Upon adoption of the ASEAN Pharmaceutical Regulatory Framework Agreement which is legally binding [33], the subsequent step is to develop the ASEAN Pharmaceutical Regulatory Framework (APRF). APAC aims to contribute as interested parties on the scientific, regulatory and technical arm of APRF. Collectively, APAC may also consider ways to support a regional reliance program coordinated for multiple regulators to reach their regulatory decision around the same time [2].

### Advancing Digital Transformation

With an increasingly fast digitalization and shifting to sustainable operation models, strong partnership between NRAs and APAC can expedite the adoption of telehealth and digital technologies ushered in by COVID-19 [34]. APAC is committed to working with key stakeholders including our NRAs to sustain and enable a new era of innovation to benefit patients without compromising the robust standard of approval.

## Figures and Tables

**Fig. 6:**
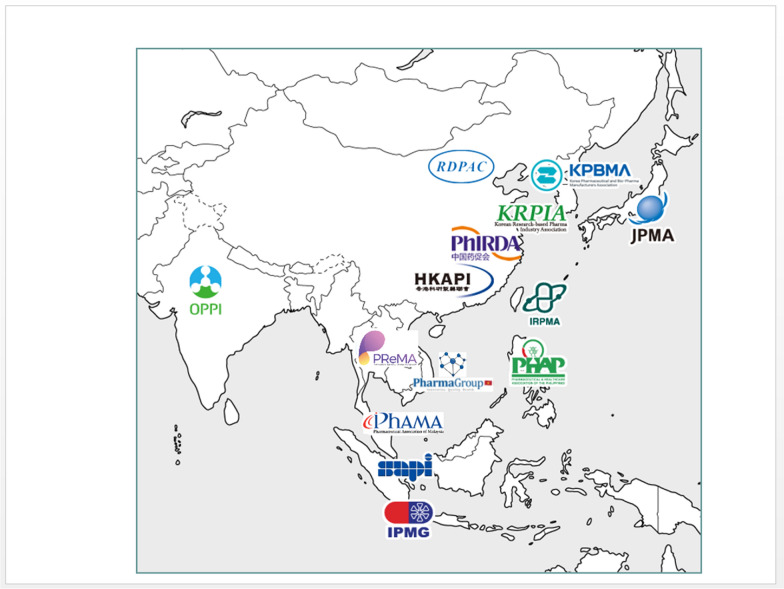
Member associations from 12 economies

## Appendix A

**Table:** APAC member associations:

HKAPI	The Hong Kong Association of the Pharmaceutical Industry
IPMG	International Pharmaceutical Manufacturers Group
IRPMA	International Research-based Pharmaceutical Manufacturers Association
JPMA	Japan Pharmaceutical Manufacturers Association
KPBMA	Korea Pharmaceutical and Bio-Pharma Manufacturers Association
KRPIA	Korean Research-based Pharmaceutical Industry Association
OPPI	Organization of Pharmaceutical Producers of India
PhAMA	Pharmaceutical Association of Malaysia
Pharma Group	PG Pharma Group Vietnam
PHAP	Pharmaceutical and Healthcare Association of the Philippines
PhIRDA	China Pharmaceutical Innovation & Research Development Association
PReMA	Pharmaceutical Research & Manufacturers Association
RDPAC	R&D-based Pharmaceutical Association Committee
SAPI	Singapore Association of Pharmaceutical Industries

In addition, PG Pharma Group Vietnam participates in the activities of APAC Regulations and Approvals Expert Working Group (RA-EWG) as an observer.

See Fig. 6.

## Abbreviations

2D: Two-dimensional
AJA: ASEAN Joint Assessment
APAC: Asia Partnership Conference of Pharmaceutical Association
APRF: ASEAN Pharmaceutical Regulatory Framework
APRP: ASEAN Pharmaceutical Regulatory Policy
ASEAN: Association of South East Asian Nations
DP: Drug Product
DS: Drug Substance
eCPP: Electronic Certificate of Pharmaceutical Products
eGMP: Electronic GMP
EMA: European Medicines Agency
ePI: Electronic product information
EUA: Emergency Use Authorization
FAQ: Frequently Asked Questions
FDA: Food and Drug Administration
GMP: Good Manufacturing Practice
ICH: International Council for Harmonization of Technical Requirements for Pharmaceuticals for Human Use
IFPMA: International Federation of Pharmaceutical Manufacturers & Associations
ISO/IEC: International Organization for Standardization / International Electrotechnical Commission
JACG: Joint Assessment Coordinating Group
MOH: Ministry of Health
MRA: Mutual Recognition Arrangement
NRAs: National Regulatory Authorities
NPRA: National Pharmaceutical Regulatory Agency
OPPI: Organization of Pharmaceutical Producers of India
PACMP: Post Approval Change Management Protocol
PhAMA: Pharmaceutical Association of Malaysia
PIC/S: Pharmaceutical Inspection Co-operation Scheme
R&D: Research and Development
RA-EWG: Regulations and Approvals Expert Working Group
RWD/E: Real World Data/Evidence
WHO: World Health Organization

## Footnotes

1 APAC is an industry-driven initiative led by R&D-based pharmaceutical associations from Asian economies, aiming to fulfill its mission. https://apac-asia.com/. A list of member associations is available as an appendix.

2 https://apac-asia.com/images/achievements/pdf/10th/APAC%20letter%20related%20to%20COVID-19.pdf

3 10th APAC Virtual Conference Theme: Overcoming COVID-19 and Taking on New Innovative Challenges for the Next Decade in Asia.(2021); https://apac-asia.com/achievements/10th_apac.html.

4 APAC Thanks Letter Related to Covid-19 (2021). https://apac-asia.com/images/achievements/pdf/10th/APAC Thanks Letter related to COVID-19.pdf.

5 Progress-Status Feedback by each association_Final (002).xlsx (live.com)

6 Data Collection into the Simplified Progress Feedback Table (2021); https://apac-asia.com/images/achievements/pdf/11th/SimplifiedProgressfeedbackTable.pdf.

7 Reply from Taiwan FDA in Response to APAC Letter related to COVID-19 (2020); https://apac-asia.com/images/achievements/pdf/11th/2020Aug_Official%20Reply%20from%20Taiwan%20FDA.pdf

8 Pharmaceutical Market & Regulatory Environment in Asia (PRME) Analysis Report for Year 2020 & 2021; https://apac-asia.com/groups/ra/publications.html.
